# Transduction of Cu, Zn-superoxide dismutase mediated by an HIV-1 Tat protein basic domain into human chondrocytes

**DOI:** 10.1186/ar1972

**Published:** 2006-06-22

**Authors:** Hyun Ah Kim, Dae Won Kim, Jinseu Park, Soo Young Choi

**Affiliations:** 1 Department of Internal Medicine, Hallym University Sacred Heart Hospital, 896, Pyongchondong, Dongan-gu, Anyang, Kyunggi-do, 431-070, Korea; 2 Department of Biomedical Sciences and Research Institute for Bioscience and Biotechnology, Hallym University, Chunchon 200-702, Korea

## Abstract

This study was performed to investigate the transduction of a full-length superoxide dismutase (SOD) protein fused to transactivator of transcription (Tat) into human chondrocytes, and to determine the regulatory function of transduced Tat-SOD in the inflammatory cytokine induced catabolic pathway. The pTat-SOD expression vector was constructed to express the basic domain of HIV-1 Tat as a fusion protein with Cu, Zn-SOD. We also purified histidine-tagged SOD without an HIV-1 Tat and Tat-GFP as control proteins. Cartilage samples were obtained from patients with osteoarthritis (OA) and chondrocytes were cultured in both a monolayer and an explant. For the transduction of fusion proteins, cells/explants were treated with a variety of concentrations of fusion proteins. The transduced protein was detected by fluorescein labeling, western blotting and SOD activity assay. Effects of transduced Tat-SOD on the regulation of IL-1 induced nitric oxide (NO) production and inducible nitric oxide synthase (iNOS) mRNA expression was assessed by the Griess reaction and reverse transcriptase PCR, respectively. Tat-SOD was successfully delivered into both the monolayer and explant cultured chondrocytes, whereas the control SOD was not. The intracellular transduction of Tat-SOD into cultured chondrocytes was detected after 1 hours, and the amount of transduced protein did not change significantly after further incubation. SOD enzyme activity increased in a dose-dependent manner. NO production and iNOS mRNA expression, in response to IL-1 stimulation, was significantly down-regulated by pretreatment with Tat-SOD fusion proteins. This study shows that protein delivery employing the Tat-protein transduction domain is feasible as a therapeutic modality to regulate catabolic processes in cartilage. Construction of additional Tat-fusion proteins that can regulate cartilage metabolism favorably and application of this technology in *in vivo *models of arthritis are the subjects of future studies.

## Introduction

Osteoarthritis (OA) is a degenerative disease of articular cartilage and causes significant morbidity in humans. It is characterized by loss of articular cartilage matrix, mainly collagen and proteoglycans, leading to tissue destruction and cell death, eventually resulting in loss of joint function. Although OA is frequently regarded as a noninflammatory form of arthritis, considerable data implicate proinflammatory cytokines derived from both the synovium and the chondrocytes in cartilage destruction. IL-1 and its downstream mediators, such as inducible nitric oxide synthase (iNOS), cyclooxygenase 2 (COX-2) and phospholipase A_2_, are postulated as the important factors in the inflammatory cascade of OA [1]. Also, IL-1 has been shown to induce chondrocytes to produce the reactive oxygen species (ROS), which act as second messengers in the intracellular signaling pathways involved in activation of proinflammatory responses and mediate degradation of aggregan and collagen [2]. Overproduction of the ROS also causes apoptosis and necrosis, resulting in cellular damage [3]. Cell defense against the ROS utilizes antioxidant enzymes such as superoxide dismutase (SOD), catalase, and glutathione peroxidase [4]. SOD catalyzes the breakdown of superoxide to generate hydrogen peroxide. Catalase and glutathione peroxidases are two cellular defenses that serve to remove hydrogen peroxide by decomposing it into water and oxygen and to reduce generation of hydroxyl radicals [5]. Therefore, these enzymes are vital for maintaining a balanced cellular redox state and therapeutic manipulation of them is thought to have a role in combating the toxic effects of oxygen free radical induced damage.

A variety of therapeutic strategies for OA have been developed to antagonize the activity of proinflammatory cytokines. These strategies include gene delivery techniques, such as *ex vivo *gene transfer of glucuronosyltransferase-I [6], retrovirus expression of IL-1 receptor antagonists [7], and adenovirus-mediated overexpression of transforming growth factor-β [8]. Although gene therapy has been considered a promising method for introducing therapeutic molecules into cells, this technique bears significant constraints, such as efficacy of gene delivery, duration of gene expression and toxicity. Previously, it has been reported that the basic domain of the HIV-1 transactivator of transcription (Tat) protein, which is composed of 11 amino acid residues, possesses the ability to traverse biological membranes efficiently in a process termed 'protein transduction' [9,10]. The HIV-1 Tat protein transduction domain (PTD), in common with similar domains found in VP22 from the herpes simplex virus [11] and Antennapedia from *Drosophila *[12], is a region rich in positively charged amino acids that are thought to interact with negatively charged phospholipids in the mammalian plasma membrane. The transduction occurs by a receptor- or transporter-independent fashion that appears to target the lipid bilayer directly [13]. HIV-1 Tat proteins thus have been shown to serve as carriers that direct uptake of various heterologous proteins into cells *in vitro *and *in vivo*. Recently, this property has been used in therapeutic applications. In this study, we showed that the full-length SOD protein fused to HIV-1 Tat PTD is transduced into human chondrocytes, both in monolayer and explant cultures, and that the transduced fusion protein has a regulatory function for the IL-1 induced catabolic pathway in chondrocytes.

## Materials and methods

### Reagents

Recombinant human IL-1β was obtained from R&D systems (Minneapolis, MN, USA). Anti-polyhistidine IgG was purchased from Santa Cruz Biotechnologies (Santa Cruz, CA, USA). Nitrate/nitrite colorimetric assay kits were purchased from Cayman Chemical (Ann Arbor, MI, USA). Restriction endonucleases and T4 DNA ligase were purchased from Promega (Madison, WI, USA). *Pfu *polymerase was obtained from Stratagene (La Jolla, CA, USA). TA-cloning vector was obtained from Invitrogen (Carlsbad, CA, USA). Oligonucleotides were synthesized from Gibco BRL custom primers (Carlsbad, CA, USA). Isopropyl-β-D-thiogalactoside (IPTG) was obtained from Duchefa (Haarlem, the Netherlands). Plasmid pET15b and *Escherichia coli *strain BL21 (DE3) were from Novagen (Madison, WI, USA). Ni^2+^-nitrilotriacetic acid Sepharose superflow was purchased from Qiagen (Hilden, Germany). A human Cu, Zn-SOD cDNA fragment was isolated using the PCR technique from the λ_Zap _human placenta cDNA library [14] and a monoclonal antibody against human Cu, Zn-SOD was produced in our laboratory. All other reagents were obtained from Sigma (St. Louis, MO, USA) unless specified otherwise.

### Expression and purification of Tat-SOD

The pTat-SOD expression vector was constructed to express the basic domain (amino acids 49 to 57) of HIV-1 Tat as a fusion protein with Cu, Zn-SOD, as described previously [15]. Briefly, two oligonucleotides were synthesized and annealed to generate a double-stranded oligonucleotide encoding nine amino acids from the basic domain of HIV-1 Tat. The sequences were: top strand, 5'-TAGGAAGAAGCGGAGACAGCGACGAAGAC-3'; and bottom strand, 5'-TCGAG-TCTTCGTCGCTGTCTCCGCTTCTTCC-3'. The double-stranded oligonucleotide was directly ligated into the *Nde*I-*Xho*I-digested pET15b in frame with the six histidine open-reading frames to generate the HisTat expression plasmid, pHisTat. Next, on the basis of the cDNA sequence of human Cu, Zn-SOD, two oligonucleotides were synthesized. The top strand, 5'-CTCGAGGCGACGAAGGCCGTGTGCGTG-3', contained a *Xho*I restriction site, and the bottom strand, 5'-GGATCCTTATTG-GGCGATCCCAATTAC-3', contained a *Bam*HI restriction site. The reaction mixture was made up in a 50 μl siliconized reaction tube and heated at 94°C for 5 minutes. The program for PCR consisted of 30 cycles of denaturation at 94°C for 40 seconds, annealing at 54°C for 1 minute, and elongation at 70°C for 3 minutes, and the final extension at 72°C for 10 minutes. The PCR products were purified by preparative agarose gel electrophoresis. The purified products were ligated into a TA-cloning vector and then transformed into a competent cell. The bacterial cells (*E. coli *BL21) transformed with pTat-SOD were harvested and disrupted by sonication in 5 ml binding buffer (5 mM imidazole, 0.5 M NaCl, 20 mM Tris-HCl, pH 7.9) containing 6 M urea. After centrifugation, supernatant was immediately loaded onto a 2.5 ml Ni^2+^-nitrilotriacetic acid Sepharose column (Qiagen, Valencia, CA, USA). After the column was washed with 10 volumes of a binding buffer and 6 volumes of a washing buffer (60 mM imidazole, 0.5 M NaCl, 20 mM Tris-HCl, pH 7.9), the fusion protein was eluted with an elution buffer (1 M imidazole, 0.5 M NaCl, 20 mM Tris-HCl, pH 7.9). The fusion protein containing fractions were combined and the salts were removed using PD10 column chromatography. To enhance the transduction efficacy of Tat-SOD, copper ion recovery of overexpressed Tat-SOD was conducted as described previously [16]. We purified six histidine residue-tagged SODs without an HIV-1 Tat PTD by using a SOD expression vector (pSOD) as a control SOD protein. pTat-green fluorescent protein (GFP) was also constructed to express the basic domain of HIV-1 Tat as a fusion with GFP as was described previously [17] and used as a control Tat protein.

### Chondrocyte culture

Cartilage samples were obtained from the femoral condyle and tibial plateau of OA patients at the time of joint replacement surgery. All cartilage samples were procured after obtaining oral informed consent from the patients and institutional approval. Chondrocytes from articular cartilage were cultured in monolayer as described previously [18]. After about seven days, confluent chondrocytes were split once, plated and these first passage adherent chondrocytes were used in subsequent experiments. For cartilage explant culture, full-thickness cartilage slices were obtained above the subchondral bone from a relatively lesion-free area of the femoral condyle of OA patients. Each slice was cut further and a piece of approximately 2 mm width by 5 mm length by full thickness was cultured in a 48-well culture plate in 200 μl per well of the same medium in which monolayer chondrocytes were cultured. Explants were incubated in the medium for three days before protein transduction to allow them to stabilize in *in vitro *conditions.

### Transduction of Tat-SOD into chondrocytes

For the transduction of Tat-SOD protein into the monolayer cultured chondrocytes, cells were seeded at 5 × 10^5^/well in six well plates. Then, the culture medium was replaced with 1 ml of fresh serum free DMEM containing various concentrations of fusion protein. For the transduction of Tat-SOD into the cartilage explant culture, the culture medium was replaced with 200 μl of fresh serum free DMEM containing various concentrations of fusion proteins. The transduction procedures were the same for control SOD and Tat-GFP proteins.

### Western blot analysis

Monolayer cultured chondrocytes were extensively washed after protein transduction, and trypsinized. Cellular proteins were extracted in lysis buffer containing 50 mM sodium acetate, pH 5.8, 10% v/v SDS, 1 mM EDTA, 1 mM phenylmethylsulfonyl fluoride, and 1 μg/ml aprotinin at 4°C. Western blotting was performed as described previously [18], using a rabbit anti-polyhistidine IgG (Santa Cruz Biotechnologies; dilution 1:500), the epitope of which is specific for the polyhistidine domain of Tat-SOD and control SOD. For explant-cultured chondrocytes, tissues were milled in liquid nitrogen after protein transduction and extensive washing. Protein was extracted in 4 M guanidine hydrochloride buffer containing 50 mM sodium acetate, pH 5.8, 1 mM phenylmethylsulfonyl fluoride, and 1 μg/ml aprotinin at 4°C and dialyzed. Electrophoresis and western blotting procedures were the same as those for monolayer chondrocytes.

### SOD enzyme assay

Proteins extracted from the monolayer chondrocytes were used for the analysis of dismutase activity of SOD. The inhibition of ferricytochrome c reduction by the xanthine/xanthine oxidase reaction was monitored, according to a method reported previously [19]. The standard assay was performed in 3 ml of 50 mM potassium phosphate buffer at pH 7.8 containing 0.1 mM EDTA in a cuvette at 25°C. The reaction mixture contained 10 μM ferricytochrome c, 50 μM xanthine and sufficient xanthine oxidase to produce a rate of reduction of ferricytochrome c at 550 nm of 0.025 absorbance unit per minute. Under these defined conditions, the amount of superoxide dismutase required to inhibit the rate of reduction of ferricytochrome c by 50% is defined as 1 unit of activity.

### Fluorescence analysis and immunohistochemistry

For direct detection of transduced SOD protein in monolayer cultured chondrocytes, purified Tat-SOD and control SOD were labeled with FITC using an EZ-Label FITC protein labeling kit according to the manufacturer's instructions (Pierce, Rockford, IL, USA). Chondrocytes were seeded on glass coverslips and treated with 3 μM Tat-SOD or control SOD proteins. After incubation for 1 hour at 37°C, the cells were washed extensively with PBS. The distribution of fluorescence in non-fixed cells was analyzed with a Carl Zeiss Axiophot fluorescence microscope (Oberkochen, Germany). Immunohistochemical studies were carried out to identify transduced Tat-SOD in cartilage explant cultures. The cartilage explants were fixed with 4% paraformaldehyde after protein transduction and extensive washing. After fixation, explants were stored in 30% sucrose in PBS, and then cut in a cryotome. After removal of non-specific immunoreactivity using 0.3% Triton X-100 and 10% normal rabbit serum in PBS, immunohistochemistry was performed using a mouse monoclonal anti-human Cu, Zn-SOD IgG (dilution 1:500). The presence and distribution of Cu, Zn-SOD were determined by a confocal scanning fluorescent microscope (LSM510, Zeiss).

### Nitrate/nitrite quantification

The final products of NO *in vivo *are nitrite and nitrate, the sum of which can be used as an index of total NO production. Chondrocytes were transduced with Tat-SOD protein, control SOD protein or Tat-GFP protein. After transduction, cells were extensively washed, and fresh serum free DMEM with or without IL-1 (1 ng/ml) was replenished. Culture media were harvested after 24 hours, and then analyzed using a nitrate/nitrite colorimetric assay kit, as recommended by the manufacturer. Briefly, nitrate was converted to nitrite using nitrate reductase, and then the Griess reagents were added to form a deep-purple azo compound. Absorbance was measured at 540 nm using a plate reader to determine nitrite concentrations. The detection limit of the assay was 1 μM.

### Reverse transcriptase-PCR

After transduction of monolayer chondrocytes, fresh serum free DMEM with or without IL-1 (1 ng/ml) was replenished. Total RNA was isolated from chondrocytes after 4 hours using the RNeasy Mini Kit (Qiagen, Valencia, CA, USA). Total RNA (200 ng) was reverse transcribed using the SuperScript™ First-strand synthesis system for reverse transcriptase (RT)-PCR (Invitrogen, Gaithersburg, MD, USA) with oligo(dT)_20 _primers. PCR amplification of cDNA aliquots was performed with the following sense and antisense primers (5'→3'): iNOS sense, GTG AGG ATC AAA AAC TGG GG; iNOS antisense, ACC TGC AGG TTG GAC CAC; glyceraldehyde 3-phosphate dehydrogenase (GAPDH) sense, CGA TGC TGG GCG TGA GTA C; and GAPDH antisense, CGT TCA GCT CAG GGA TGA CC. Reactions were processed through 25 cycles of 30 seconds of denaturation at 94°C, 1 minute of annealing at 55°C for GAPDH and 30 cycles of 45 seconds of denaturation at 95°C, 30 seconds of annealing at 55°C for iNOS, followed by 1 minute of elongation at 72°C. PCR conditions were chosen to be non-saturating in all cases. PCR products were run on a 1.5% agarose gel, stained with ethidium bromide and visualized using a UVP transilluminator(Ultraviolet Product, Upland, CA). The band densities were quantified using the National Institues of Health,(NIH) image program (Bethesda, MD).

### Data analysis

Data are expressed as means ± standard deviations. Differences between treatment groups were tested by using the Mann-Whitney *U *test (GraphPad Prism, version 3, GraphPad Software, San Diego, CA, USA). Significance was established at the 95% confidence level (*p *< 0.05).

## Results

### Transduction of Tat-SOD into monolayer cultured chondrocytes

**Figure 1:**
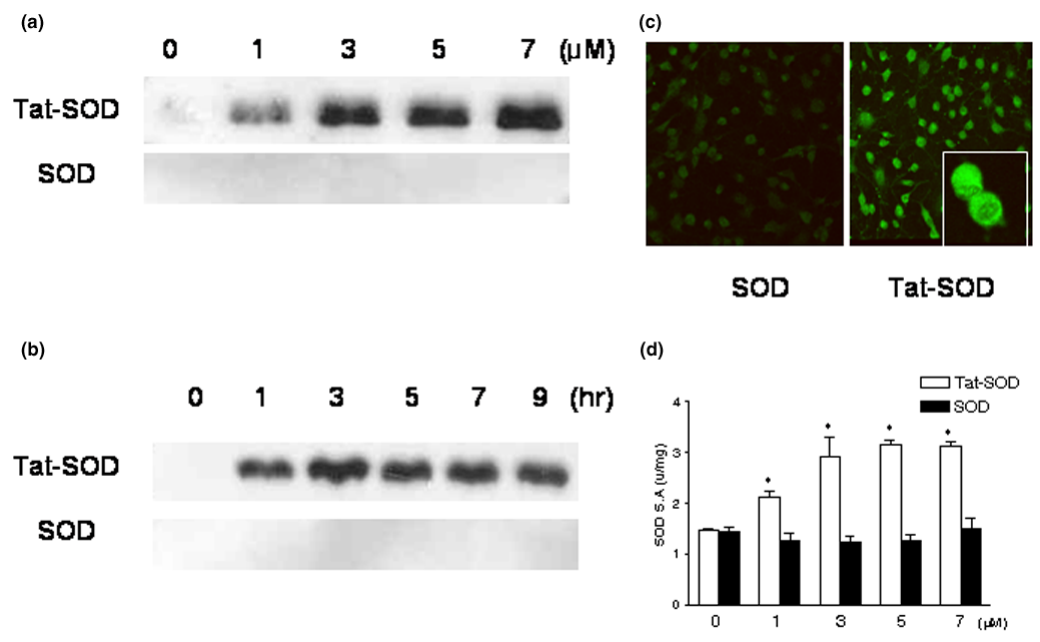
Transduction of transactivator of transcription (Tat)-superoxide dismutase (SOD) fusion protein into monolayer cultured chondrocytes. Chondrocytes were obtained from the femoral condyle and the tibial plateau from osteoarthritis (OA) patients and cultured in monolayers. First passage chondrocytes were used in subsequent experiments. **(a) **Dose-dependent and **(b) **time-dependent transduction of Tat-SOD into chondrocytes. Transduction of Tat-SOD into the cells was analyzed by western blotting with a rabbit anti-polyhistidine IgG. Tat-SOD (1 to 7 μM) and control SOD were added to the culture medium for 1 hour (a), or 3 μM of Tat-SOD and control SOD were added to the culture medium for 1 to 9 hours (b). **(c) **Localization of transduced Tat-SOD protein. After FITC-labeled Tat-SOD (3 μM) was transduced into chondrocytes, the cells were washed with PBS and immediately observed by fluorescence microscopy (×100 original magnification; inset, ×400 original magnification). Data are representative of four samples from different donors. **(d) **The specific activities of SOD in cultured chondrocytes treated with Tat-SOD; 1 to 7 μM of Tat-SOD and control SOD were added to the culture medium for 1 hours. Bars represent the mean ± standard error of the mean obtained from duplicate experiments from three donors. Asterisks denote *p *< 0.05 compared to control.

To determine whether Tat-SOD fusion protein is able to traverse the membrane of cultured chondrocytes, we added a variety of concentrations (1 to 7 μM) of fusion protein to the culture media for 1 hour, and then determined the levels of protein transduced into the cells by western blotting. Neither Tat-SOD nor control SOD fusion proteins were cytotoxic to chondrocytes in the concentration range employed in our experiment (data not shown). As shown in Figure 1a, Tat-SOD was detected in chondrocyte lysates, whereas control SOD was not. The transduction of Tat-SOD into cultured cells was maximal at 3 μM and did not increase further by increasing the concentration of the fusion protein. To determine the time-dependence of Tat-SOD delivery into chondrocytes, 3 μM of Tat-SOD protein was added to the culture media of monolayer chondrocytes for 1 to 9 hours and the level of transduced proteins was analyzed by western blotting. The transduction of Tat-SOD into cultured chondrocytes was detected after 1 hour and the amount of transduced protein did not increase further by increasing the duration of incubation (Figure 1b). This result suggests that Tat-SOD was rapidly transduced into the monolayer chondrocytes. The intracellular delivery of Tat-SOD into cultured chondrocytes was further confirmed by direct fluorescence analysis. Monolayer cultured chondrocytes were found to be transduced with Tat-SOD, whereas only faint fluorescence signals similar to that of the background were detected in cells treated with control SOD (Figure 1c). The transduction efficiency was 91.6 ± 3.7% for Tat-SOD concentration of 3 μM after 1 hour. The transduction efficiency for Tat-GFP was similar to that for Tat-SOD (88.1 ± 9.1%; data not shown). The fluorescence signals detected in the chondrocytes were largely in the cytoplasm, with a minority of cells showing nuclear distribution. The restoration of authentic properties of the transduced protein in cells is a key issue in the application of protein transduction technology for therapeutic use. Therefore, we determined the dismutase activity of SOD in the chondrocytes treated with Tat-SOD and compared it with those treated with control SOD; SOD enzyme activity increased with the transduction of fusion protein into chondrocytes (Figure 1d). The intracellular dismutase activity of SOD significantly increased after treatment with 1 μM Tat-SOD for 1 hour. These data demonstrate that Tat-SOD fusion proteins were efficiently transduced into monolayer cultured chondrocytes and the delivered protein maintained its properties.

### Transduction of Tat-SOD into cartilage explant culture

**Figure 2:**
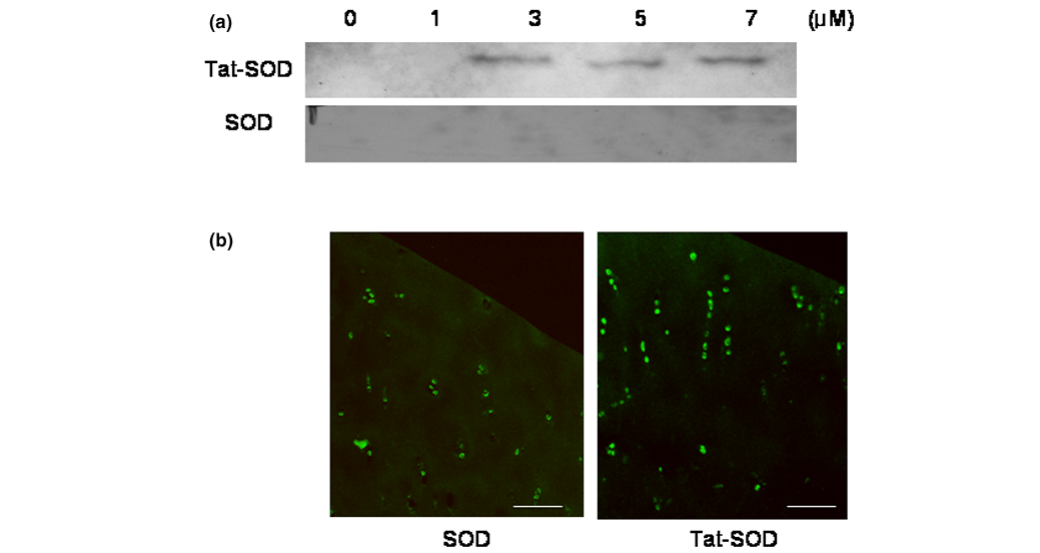
Transduction of transactivator of transcription (Tat)-superoxide dismutase (SOD) fusion protein into explant cultured chondrocytes. Chondrocytes were obtained from a relatively lesion-free area of femoral condyles in OA patients and cultured in explants. **(a) **Transduction of Tat-SOD into the cells was analyzed by western blotting with a rabbit anti-polyhistidine IgG. Tat-SOD (1 to 7 μM) or control SOD were added to the culture medium for 6 hours. The tissues were milled in liquid nitrogen after transduction and protein was extracted in 4 M guanidine hydrochloride buffer and dialyzed. **(b) **Immunohistochemistry of cartilage explant transduced with Tat-SOD fusion protein (3 μM concentration of fusion protein added to the culture medium for 6 hours). The explants were fixed and sectioned in a cryotome after extensive washing. Cartilage sections were incubated with a mouse monoclonal anti-human Cu, Zn-SOD IgG and then visualized with a confocal scanning fluorescent microscope. Data are representative of seven samples from different donors. Scale bar = 100 μm.

Next, we tried to transduce the Tat-SOD proteins into chondrocytes embedded in the extracellular matrix of cartilage. Human cartilage explants were treated with 1 to 7 μM of fusion protein for 6 hours, and the levels of protein transduced into the cells were analyzed by western blotting of protein extracted from the explants. Faint but definite bands of transduced protein probed by a rabbit anti-polyhistidine IgG were detected in lysates from Tat-SOD transduced chondrocytes, starting from a concentration of 3 μM (Figure 2a). Next, immunohistochemistry was performed with a mouse monoclonal anti-human Cu, Zn-SOD IgG in cartilage slices treated with 3 μM of Tat-SOD or control SOD; Cu, Zn-SOD was detected in the explant culture with both treatments (Figure 2b). While the intensity of staining tended to be stronger in Tat-SOD treated explants, especially near the cut surface, there was no significant difference in the percentage of positive chondrocytes between Tat-SOD and control SOD treated explants (data not shown), implying significant background expression of Cu, Zn-SOD in human OA cartilage.

### Effects of transduced Tat-SOD on IL-1 induced NO production and iNOS mRNA upregulation of chondrocytes

**Figure 3:**
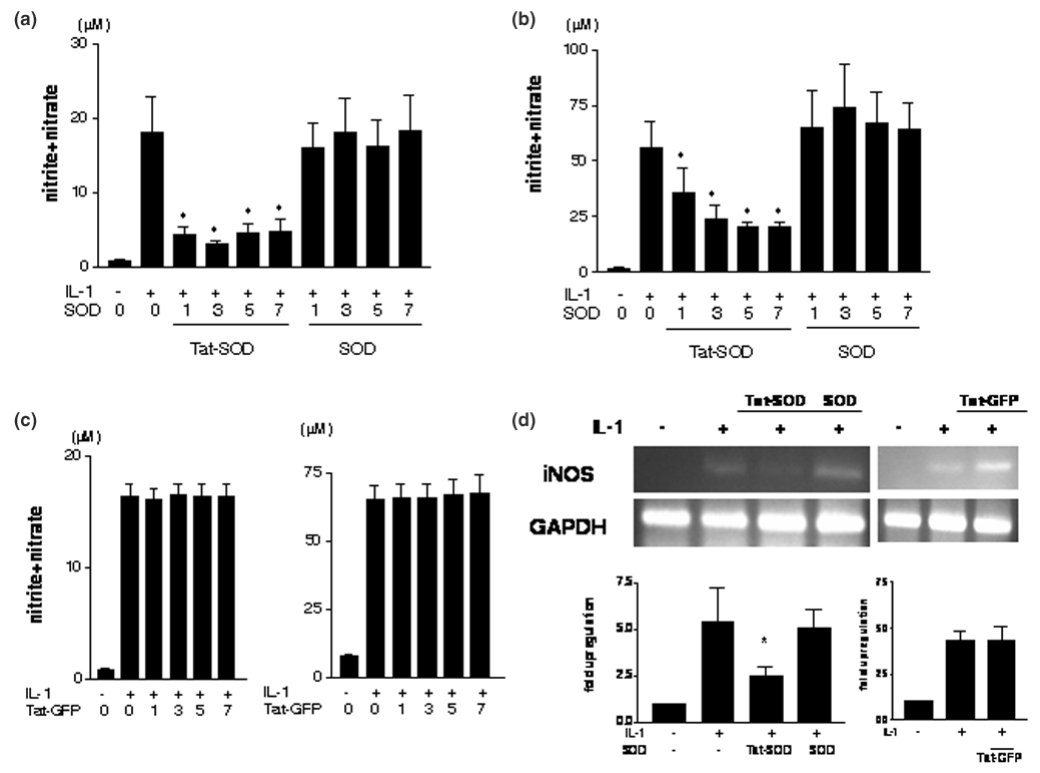
Regulation of nitric oxide and inducible nitric oxide synthase mRNA expression by transduced Tat-SOD. **(a) **After monolayer cultured chondrocytes were transduced with 1 to 7 μM of Tat-SOD, control SOD or Tat-GFP proteins, serum free DMEM with or without IL-1 (1 ng/ml) was replenished. Culture media were harvested after 24 hours, and the production of nitric oxide(NO) was analyzed using a nitrate/nitrite colorimetric assay kit. The data are means and standard deviations of duplicate experiments from samples from three different donors. Asterisks denote *p *< 0.05 compared to control (IL-1 treatment alone). **(b) **Explant cultured chondrocytes were transduced with 1 to 7 μM of Tat-SOD, control SOD or Tat-GFP proteins, and serum free DMEM with or without IL-1 (1 ng/ml) was replenished. Culture media were harvested after 72 hours, and the production of NO was analyzed using a nitrate/nitrite colorimetric assay kit. The data are means and standard deviations of duplicate experiments from samples from three different donors. Asterisks denote *p *< 0.05 compared to control (IL-1 treatment alone). **(c) **Regulation of IL-1 stimulated NO production in chondrocytes by transduced Tat-GFP was observed in monolayer (left panel) and explant (right panel) cultured chondrocytes. Chondrocytes were transduced with 1 to 7 μM of Tat-GFP, and serum free DMEM with or without IL-1 (1 ng/ml) was replenished. Culture media were harvested after 24 hours for monolayer or 72 hours for explants, and the production of NO was analyzed using a nitrate/nitrite colorimetric assay kit. The data are means and standard deviations of duplicate experiments from samples from three different donors. **(d) **After transduction of chondrocytes with 3 μM of each fusion protein, serum free DMEM with or without IL-1 (1 ng/ml) was replenished. Total RNA was isolated from chondrocytes after 4 hours and RT-PCR was performed. Data are representative of four samples from different donors. The band densities for iNOS mRNA were quantified, the percent GAPDH density was calculated for iNOS and the value for the control culture was set at 1. Asterisks denote *p *< 0.05.

To determine whether the transduced fusion protein was functionally active in the cells, we examined the effect of Tat-SOD transduction on NO production induced by IL-1. As shown in Figure 3a,b, both monolayer and explant chondrocytes produced significant NO after stimulation with 1 ng/ml IL-1. This NO production was significantly down-regulated by pretreatment with Tat-SOD fusion proteins, while control SOD failed to reduce IL-1 induced NO production, indicating that transduced Tat-SOD has a specific effect on down-regulation of inflammatory signaling. Transduction with Tat-SOD also resulted in significant down-regulation of iNOS mRNA transcription observed after treatment of chondrocytes with IL-1 (Figure 3d). To demonstrate that internalization of the Tat moiety itself has no effect on chondrocytes, Tat-GFP in addition to control SOD was used as a negative control. Tat-GFP failed to reduce IL-1 induced NO production or iNOS mRNA in chondrocytes (Figure 3c,d).

## Discussion

In this study, the transduction of the full-length SOD protein fused to HIV-1 Tat PTD into the cultured chondrocytes was examined. Non-specific results were strictly excluded by inclusion of a negative control SOD protein that carried six histidine residues, the same as Tat-SOD, without an HIV-1 Tat PTD. After extensive washing and stringent processing, the SOD signal above background was detected in none of the control SOD treated samples. In addition, Tat-GFP protein was included to demonstrate that internalization of the Tat moiety itself was not responsible for our results. Because chondrocytes are embedded in thick extracellular matrix, it is considered a challenge to deliver foreign genes or proteins into chondrocytes in cartilage. However, our findings show delivery of fusion proteins into the cartilage explant culture, demonstrating that protein delivery employing Tat-PTD is a feasible therapeutic approach for regulation of cartilage degeneration.

In our study, transduction of Tat-SOD into chondrocytes effectively inhibited the production of the proinflammatory mediator NO, which is induced by IL-1, both in monolayer and explant cultures. However, Tat-SOD transduction failed to inhibit Metalloproteinase (MMP)-1, -3 and -13 expression or prostaglandin (PG) E_2 _activation induced by the same cytokine (data not shown). A prior study of chondrocytes showed that treatment of chondrocytes with IL-1 induces ROS, which includes both hydrogen peroxide and superoxide, but that only superoxide mediated IL-1 induced NF-kB activation and iNOS expression [20]. The investigators did not report whether IL-1 induced ROS or superoxide is responsible for stimulation of MMP in chondrocytes. Based on our results, it is likely that MMP production in chondrocytes in response to IL-1 is independent of superoxide.

Several strategies are currently being developed to block the proinflammatory pathway in arthritic diseases. Chemical compounds are being developed, but it is very difficult to find a compound that will specifically block a narrow target pathway. Thus, they often lead to non-specific and widespread effects that would make their clinical application difficult. Use of decoy oligonucleotides and RNA interference technology to modulate the expression of proinflammatory genes are more specific than chemical inhibitors. However, these therapies employing gene delivery still pose more problems than solutions in terms of efficiency, safety and inflammatory and immunogenic effects elicited by viral vectors. Potential advantages in regulating cellular functions using protein transduction technology are: it allows for the application, in various pathological conditions, of our accumulated knowledge on intracellular functions of particular signaling pathways; the levels of protein inside cells are directly and specifically regulated so that maximum benefit can be achieved with minimal side effects; and it is reversible and treatment can be terminated or can resume after a resting period as deemed necessary [21]. Disadvantages include the lack of target cell specificity, potential of immunogenicity, and our incomplete knowledge of the molecular mechanisms of protein transduction. Reports of the application of PTD in potential therapeutic development are increasing in various fields and in a variety of cell systems and include inhibition of apoptosis by transduction of anti-apoptotic Bcl-_XL _protein in explant cultured human chondrocytes and in human islet cells [22,23], induction of chemosensitivity by transduction of cytosine deaminase in human tumor cells [24], and inhibition of proinflammatory signaling by transduction of superreppressor IkB in Jurkat T cells [21]. Protein transduction therapy is also showing promise in *in vivo *models, such as in the reduction of inflammation in carageenan-induced pleurisy of Wistar rat and the reduction of pancreatic islet cell toxicity in streptozotocin-induced diabetic mice [25,26].

The mechanism by which Tat mediates cell entry has been a focus of interest, and it has been shown that the entry mediated by Tat-PTD is not dependent on a specific receptor, energy generation or endocytosis [27]. Recent data provided evidence that Tat-PTD binds to the negatively charged cell surface constituents by electrostatic interaction and then stimulates membrane ruffling to form heterogeneous vesicular structures called macropinosomes [28]. It has also been reported that Tat also promotes gene transfer into mammalian cells, and may be a potential delivery vehicle for gene therapy as well [29].

A recent report shows that extracellular SOD is decreased in both human and animal models of OA, suggesting that inadequate control of reactive oxygen species plays a role in the pathophysiology of OA [30].

## Conclusion

This study demonstrates that protein delivery employing Tat-PTD is feasible as a therapeutic modality to regulate catabolic processes in cartilage. Construction of additional Tat-fusion proteins that may stimulate anabolic activity or down-regulate catabolic activity, and application to an *in vivo *model of arthritis is the subject of future studies.

## Abbreviations

DMEM = Dulbecco's modified Eagle's medium; GAPDH = glyceraldehyde 3-phosphate dehydrogenase; GFP = green fluorescent protein; IL = interleukin; iNOS = inducible nitric oxide synthase; NO = nitric oxide; OA = osteoarthritis; PBS = phosphate buffered saline; PTD = protein transduction domain; ROS = reactive oxygen species; SOD = superoxide dismutase; Tat = transactivator of transcription.

## Competing interests

The authors declare that they have no competing interests.

## Authors' contributions

HAK conceived the study, supervised the experimental procedure, and wrote the manuscript. DWK performed the experiments. JP and SYC participated in the design of the study, produced the Tat fusion protein and drafted the manuscript.
